# Real-World Administration of Once-Daily MeltDose^®^ Prolonged-Release Tacrolimus (LCPT) Allows for Dose Reduction of Tacrolimus and Stabilizes Graft Function Following Liver Transplantation

**DOI:** 10.3390/jcm10010124

**Published:** 2020-12-31

**Authors:** Katharina Willuweit, Alexandra Frey, Anne Hörster, Fuat Saner, Kerstin Herzer

**Affiliations:** 1 Department of Gastroenterology and Hepatology, University Hospital Essen, University of Duisburg-Essen, Hufelandstraße 55, 45147 Essen, Germany; alexandra.frey@uk-essen.de (A.F.); anne.hoerster@uk-essen.de (A.H.); 2 Department of General, Visceral and Transplant Surgery, University Hospital Essen, University of Duisburg-Essen, Hufelandstraße 55, 45147 Essen, Germany; fuat.saner@uk-essen.de; 3 Knappschaftsklinik Bad Neuenahr, Georg-Kreuzberg-Straße 2-6, 53474 Bad Neuenahr, Germany

**Keywords:** liver transplantation, MeltDose^®^, once-daily prolonged-release tacrolimus, LCPT, switch, efficacy, safety

## Abstract

The calcineurin inhibitor tacrolimus is included in most immunosuppressive protocols after liver transplantation. This retrospective, observational 24-month study investigated the tolerability of once-daily MeltDose^®^ prolonged-release tacrolimus (LCPT) after switching from twice-daily immediate-release tacrolimus (IR-Tac) in a real-world cohort of 150 patients with previous liver transplantation. No graft rejection or new safety signals were observed. Only 7.3% of patients discontinued LCPT due to side effects. In the overall patient population, median liver transaminases, total cholesterol, triglycerides, glucose, and HbA_1c_ remained constant after switching to LCPT. Total cholesterol significantly decreased (*p* ≤ 0.002) in patients with initially elevated levels (>200 mg/dL). A total of 71.8% of 96 patients maintained a glomerular filtration rate > 60 mL/min/1.73 m^2^ throughout the study, while 44.7% of patients were classified as fast metabolizers and 55.3% as slow metabolizers. Median daily tacrolimus dose could be reduced by 50% in fast metabolizers and by 30% in slow metabolizers, while trough levels were maintained in the target range (4–6 ng/mL). In conclusion, our observational study confirmed previous evidence of good overall tolerability and a favorable outcome for the patients after switching from IR-Tac to LCPT after liver transplantation.

## 1. Introduction

Liver transplantation is a life-saving treatment for patients with end stage liver disease and liver failure. In addition, it may cure some hereditary metabolic disorders and malignancies involving the liver in selected patients. Advances in post-transplant immunosuppression and individual tailoring of immunosuppression regimens balancing benefits and potential harm of therapy have contributed to the improvement of survival rates after liver transplantation [1].

The calcineurin inhibitor (CNI) tacrolimus is highly effective in preventing acute rejection after liver transplantation [2,3,4]. It is included in most combination regimens of immunosuppressive drugs, both early after liver transplantation and as part of long-term maintenance regimens [5,6,7]. However, due to its intra- and interindividual pharmacokinetic variability and narrow therapeutic window, frequent monitoring of drug levels and dose adjustments are mandatory. Whereas suboptimal tacrolimus levels may result in graft rejection, increased levels are associated with delayed graft function, metabolic disturbances, and other complications or with nephrotoxicity [8]. Tacrolimus metabolism is controlled by many factors, particularly by cytochrome P450 enzymes [9]. In liver transplantation, fast metabolizers (defined by a ratio of blood trough concentration normalized by the daily dose (C/D ratio) <1.09 ng/mL × 1/mg) require higher doses of tacrolimus to attain the intended trough level as compared with slow metabolizers. Higher doses of immediate-release twice-daily tacrolimus (IR-Tac) in fast metabolizers are associated with increased peak tacrolimus concentrations in achieving the same target trough as slow metabolizers [10,11]. Moreover, fast tacrolimus metabolism is considered a potential risk factor for CNI nephrotoxicity and BK-polyomavirus (BKV) infection [12,13].

To overcome drawbacks of tacrolimus, immunosuppression protocols with dose reduction, switch and elimination of the agent, as well as a combination of reduced-dose tacrolimus and mechanistic target of rapamycin (mTOR) inhibitors have been investigated [14,15]. In addition, different galenic formulations have been developed, such as the once-daily prolonged-release tacrolimus created with MeltDose^®^ technology (LCPT) [16]. Due to the decreased particle size and increased surface/volume ratio of LCPT, this galenic formulation is associated with enhanced bioavailability of tacrolimus by about 40% compared to IR-Tac, allowing for a dose reduction of up to 30% [17]. Moreover, immunosuppressive therapy with LCPT delivers smooth tacrolimus levels with significantly fewer peak and trough-to-peak fluctuations [18,19].

The aim of this study was to investigate tolerability of LCPT including renal function and metabolic parameters as well as recipient graft function and adherence to immunosuppressive therapy in a large real-world cohort of patients with previous liver transplantation.

## 2. Materials and Methods

### 2.1. Patients

Inclusion criteria were adult patients (≥18 years) after receiving a liver with stable graft function (>6 months) who were converted to Envarsus to improve the adherence to treatment (in the case of Prograf) or for suspected toxicity or low bioavailability. All patients were cared for at the interdisciplinary liver transplantation outpatient service of the university clinic of Essen, Germany. Patient care, diagnostics, treatment, and assessment of data followed routine care of these patients. Written informed consent was obtained from all patients prior to inclusion.

### 2.2. Study Design

**Figure 1:**
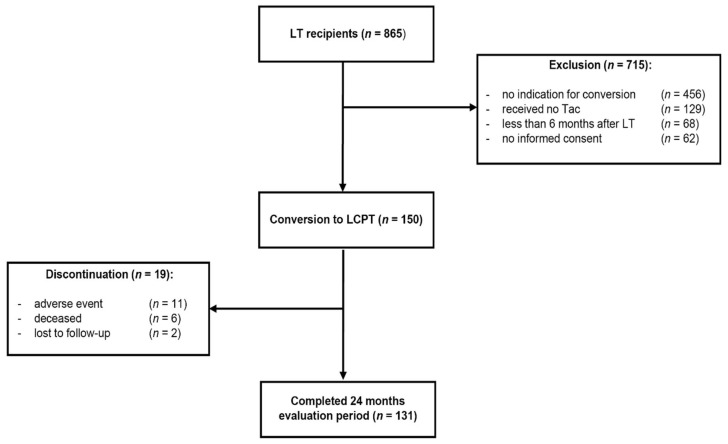
Study design. A total of 865 liver transplant (LT) recipients were screened for eligibility. During the enrolment 150 patients fulfilled the inclusion criteria and were switched to once-daily MeltDose^®^ tacrolimus (LCPT). Clinical data were analyzed in a 24-month follow-up.

This was a retrospective single-center, observational, single-arm phase 4 study. Data were from consecutive patients who visited the outpatient service within 2 years between December 2015 and December 2017. Patients, who regularly attended the aftercare clinic, were assessed, of whom 150 fulfilled the selection criteria and were included in this study (Figure 1). Patients had been on a stable dose of conventional immunosuppressive treatment with twice-daily IR-Tac (Prograf^®^, Astellas, Munich, Germany) and showed stable tacrolimus trough concentrations within the target range of 4 to 6 ng/mL. They were then switched to MeltDose^®^ LCPT (Envarsus^®^, Chiesi, Hamburg, Germany) based on a conversion factor of 1:0.7 (mg/mg), and LCPT dose was adjusted individually in order to achieve tacrolimus trough concentrations within the target range. The administration of tacrolimus formulations to the patients followed clinical criteria and did not depend on their participation in this study. Concomitant medication remained unchanged.

The primary aim of the study was to assess tolerability of LCPT and graft function within the 24-month observational period. Other endpoints included association of tacrolimus-based immunosuppression and occurrence of specific adverse events such as change of renal function, infections, or hematological alterations.

This study was conducted in compliance with independent ethics committees/institutional review boards (Ethics committee of the University Hospital Essen (AZ 16-6815-BO)), informed consent regulations, the Declaration of Helsinki (Version 2013), and International Conference on Harmonisation (ICH) Good Clinical Practice (GCP) Guidelines.

### 2.3. Assessments

Assessments included baseline patient characteristics such as age, sex, and medical history as well as indication for transplantation, date of transplantation, co-morbidities, adverse events, heart rate, and body mass index (BMI). Laboratory assessments included blood count, serum creatinine, and glomerular filtration rate (GFR) calculated by using the modification of diet in renal disease (MDRD) formula as parameters of kidney function; aspartate amino transferase (AST), alanine amino transferase (ALT), gamma-glutamyl transferase (GGT), and alkaline phosphatase (AP) as parameters of graft function; cholesterol and triglycerides as parameters of lipid metabolism; and random blood glucose and HbA1c as parameters of glucose metabolism as well as measurement of electrolytes, in particularly potassium because it is known that tacrolimus may cause an increase in potassium levels in the blood. Patients were followed up with laboratory checks weekly for the first 2 months and every 3 months afterwards. Liver biopsy was limited to those patients developing symptoms and with increased liver values.

Adherence to treatment was assessed using an extended version of the Morisky Medication Adherence Score [20,21].

The metabolic rate of tacrolimus was defined as the blood concentration normalized by the daily dose of tacrolimus (C/D ratio), expressed as ng/mL × 1/mg. Patients were classified as “fast” metabolizers or “slow” metabolizers based on their individual C/D ratio values according to the method described by Thölking et al. [13].

### 2.4. Statistical Analysis

Results are displayed as boxplots showing medians and 95% confidence intervals (CIs) of respective blood levels with respect to time as well as to normal or increased values at baseline. Median values and 95% CI ranges in brackets are shown according to the respective time points.

All values were used for statistical analysis, but for clarity outliers are not shown. Normal values or cutoffs of laboratory parameters are indicated by a dashed line. Multiple grouped variables were analyzed by repeated measures analysis of variance (ANOVA). Statistical significance was defined as a *p* value ≤ 0.05. The effect size (ES) is indicated by Cohen’s *f* Values of 0.10, 0.25, and 0.4, which are defined by Cohen as small, medium, and large ES [22].

## 3. Results

### 3.1. Patient Characteristics

A total of 150 patients were included in the study and switched to LCPT. A total of 62% of patients were male, while 38% were female (Table 1). Mean age was 55 (range, 18–77) years. About half of the patients were switched within 1–5 years after transplantation to LCPT-based immunosuppression. After liver transplantation, 22% of patients were switched within 12 months, while 27.3% of patients were switched later than five years after transplantation. At baseline, 26% of patients received mycophenolate mofetil (Roche, Basel, Swizerland) and 10% prednisolon (5 mg per day).

The most frequent indications for liver transplantation were alcoholic liver cirrhosis in 22% of patients, hepatocellular carcinoma in 21.3%, and autoimmune liver disease (primary biliary cirrhosis (PBC)/primary sclerosing cholangitis (PSC)/autoimmune hepatitis (AIH)) in 16% of patients (Table 1).

### 3.2. Patient Disposition and Adherence

Nineteen (12.7%) patients discontinued treatment with LCPT. In 11 (7.3%) patients, discontinuation was due to adverse effects between week 4 and month 18 after switching therapy. Two patients were lost to follow-up in months 6 and 12, respectively. Six patients died during the observational period.

Adherence to treatment was high throughout the study, with the mean ECS being 5 (range, 0–24) at the time of switching and 5 (range, 0–33) one year after switching to LCPT.

### 3.3. Liver Graft Function

**Figure 2:**
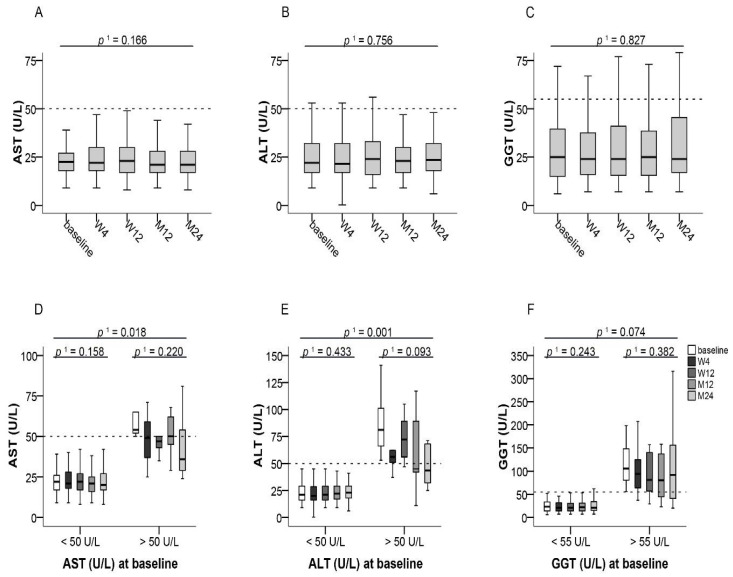
Liver transaminases after switching to LCPT. (**A**–**C**): Boxplots show median values with 95% CI for AST, ALT, and GGT at the baseline visit as well as at weeks 4 and 12 and at months 12 and 24; (**D**–**F**): Boxplots show median values with 95% CI for AST, ALT, and GGT according to normal or increased transaminase values at baseline. ^1^ with Greenhouse–Geisser correction; ALT: alanine amino transferase, AST: aspartate amino transferase, GGT: gamma-glutamyl transferase, LCPT: once-daily MeltDose^®^ prolonged-release tacrolimus, M: month, W: week.

No rejection of the liver graft was observed in any of the patients. When switching treatment, all patients had normal liver function tests except for 8.4% of patients with elevated AST, 10.7% of patients with elevated ALT, and 19.8% of patients with elevated GGT levels (Figure 2D–F). After switching from IR-Tac to LCPT, median levels of AST, ALT, and GGT remained almost unchanged and within the normal range in the whole study population during the 24-month observational period (Figure 2A–C). Patients with normal (AST: ≤50 U/L, ALT: ≤50 U/L; GGT: ≤55 U/L) or increased transaminases at the time of switching did not show any change of median transaminase levels between baseline and month 24 after switching from IR-Tac to LCPT (Figure 2D–F).

### 3.4. Kidney Function

**Figure 3:**
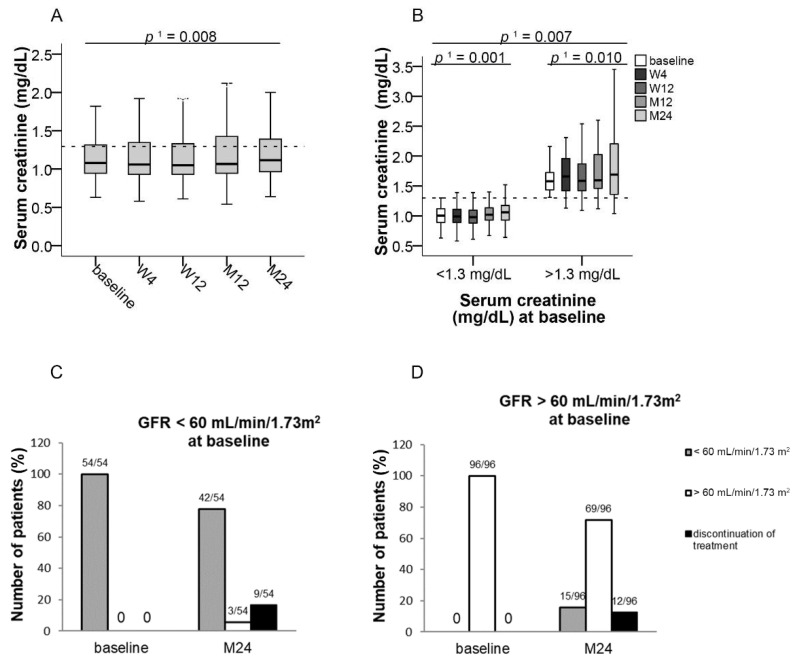
Median serum creatinine and GFR after switching to LCPT at the baseline visit as well as at weeks 4 and 12 and at months 12 and 24. Boxplots show median values with 95% CI (**A**) for serum creatinine and (**B**) for serum creatinine according to normal or increased values at baseline; (**C**,**D**) show the number of patients for GFR categories < or > 60 mL/min/1.73 m^2^ at baseline and at 24 months who initially (**C**) had a GFR < 60 mL/min/1.73 m^2^ or (**D**) had a GFR > 60 mL/min/1.73 m^2^; ^1^ with Greenhouse–Geisser correction; GFR: glomerular filtration rate, LCPT: once-daily MeltDose^®^ prolonged-release tacrolimus, M: month, W: week.

Baseline serum creatinine concentrations were normal (≤1.3 mg/dL) in 97 (74.0%) patients at the time of switching from IR-Tac to LCPT. Patients with normal serum creatinine at baseline (≤1.3 mg/dL) showed a slight increase of median serum creatinine by 0.07 from 0.99 (95% CI, 0.63–1.28) to 1.06 mg/dL (95% CI, 0.64–2.0) at month 24 (*p* ≤ 0.001, *f* = 0.23) within the normal range (Figure 2F and Figure 3B). In patients whose serum creatinine was increased at baseline (>1.3 mg/dL), a slight increase by 0.02 mg/dL of median serum creatinine from 1.57 (95% CI, 1.3–3.1) to 1.59 mg/dL (95% CI, 1.59–4.52) at month 24 (*p* ≤ 0.01, *f* = 0.11) was also observed (Figure 3B). At baseline, 54 (36.0%) patients had a reduced GFR ≤ 60 mL/min/1.73 m^2^. Of these, 5.5% experienced an improvement of kidney function to a GFR > 60 mL/min/1.73 m^2^. Reasons for treatment discontinuation in seven patients with GFR < 60 mL/min/1.73 m^2^ were asthenia, diarrhea, and musculoskeletal complaints, while one patient was lost to follow-up and four died over the course of the study (Figure 3C). Ninety-six (64.0%) patients initially showed a GFR of >60 mL/min/1.73 m^2^ at baseline. Of those, 71.8% of patients maintained a stable GFR of >60 mL/min/1.73 m^2^ until month 24. Nine patients with GFR > 60 mL/min/1.73 m^2^ discontinued treatment due to adverse events such as headache, insomnia, and hair loss, two patients died, and one patient was lost to follow-up (Figure 3D).

### 3.5. Lipid and Glucose Metabolism

**Figure 4:**
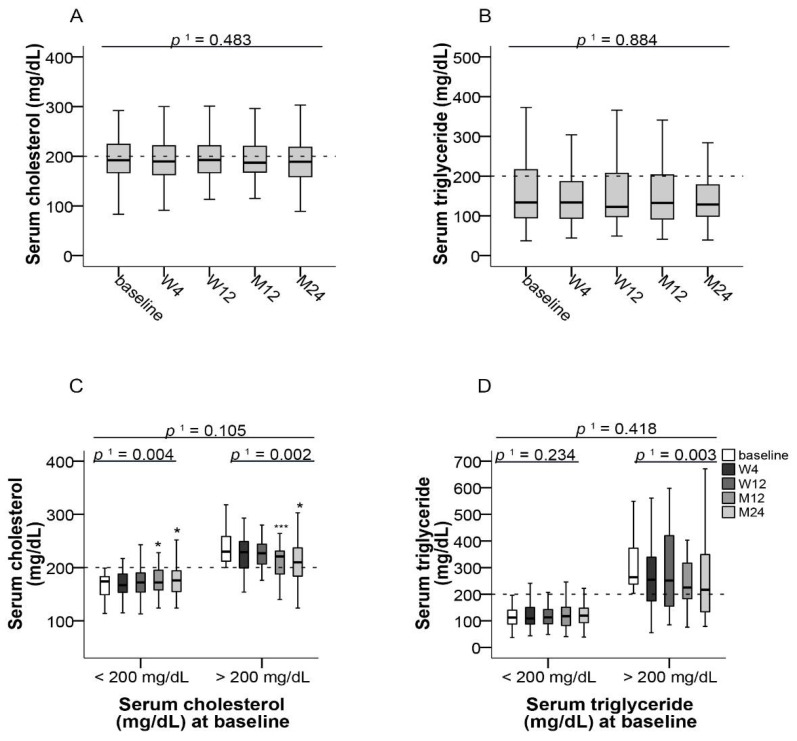
Blood lipids after switching to LCPT. (**A**) Boxplots show median values with 95% CI for cholesterol and (**B**) triglycerides at the baseline visit as well as at weeks 4 and 12 and at months 12 and 24; (**C**) Boxplots show median values with 95% CI for cholesterol and (**D**) triglycerides according to normal or increased respective values at baseline. ^1^ with Greenhouse–Geisser correction; * *p* ≤ 0.05, *** *p* ≤ 0.001; LCPT: once-daily MeltDose^®^ prolonged-release tacrolimus, M: month, W: week.

Median cholesterol and triglyceride levels remained unchanged over the study period in the whole study population (Figure 4A,B). Median cholesterol levels of all patients were 193 mg/dL (95% CI, 83–318) at baseline and 189 mg/dL (95% CI, 89–360) at month 24 (Figure 4A). Median triglyceride levels were 133 mg/dL (95% CI, 37–677) at baseline and 129 mg/dL (95% CI, 39–709) at month 24 (Figure 4B).

55.7% and 69.5% of patients had normal cholesterol and triglyceride levels (≤200 mg/dL, each), respectively, at baseline. Significant decreases of median cholesterol levels from baseline to month 24 (*p* ≤ 0.004, *f* = 0.26 and *p* ≤ 0.002, *f* = 0.46 respectively) were observed both in patients who had cholesterol concentrations ≤200 mg/dL as well as in those with >200 mg/dL at baseline (Figure 4C). No significant changes of triglyceride levels were observed in patients with normal triglyceride levels at baseline, whereas patients with increased triglyceride levels at base showed a significant reduction (*p* ≤ 0.003; Figure 4D).

**Figure 5:**
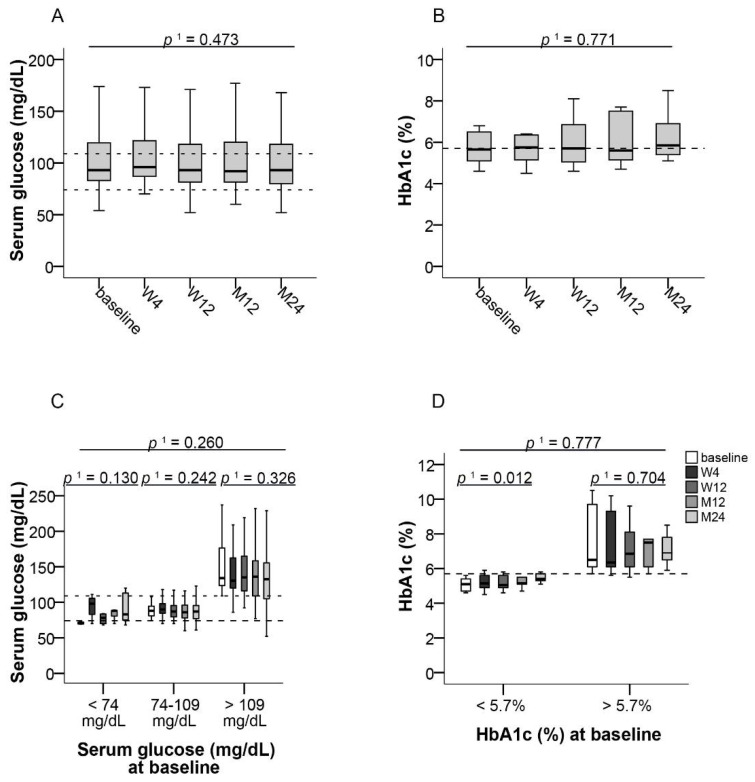
Parameters of glucose metabolism after switching to LCPT. (**A**) Boxplots show median values with 95% CI for blood glucose and (**B**) HbA_1c_ at the baseline visit as well as at weeks 4 and 12 and at months 12 and 24; Boxplots show median values with 95% CI for (**C**) blood glucose and (**D**) HbA_1c_ according to normal or increased respective values at baseline. ^1^ with Greenhouse–Geisser correction; LCPT: once-daily MeltDose^®^ prolonged-release tacrolimus, M: month, W: week.

Median blood glucose concentration of all patients remained nearly unchanged after switch to LCPT over the observational period (Figure 5A). A total of 61.8% of patients initially had blood glucose concentrations within the reference range (74–109 mg/dL) and these remained within this range during the observational period in these patients. Median blood glucose values were 88 mg/dL (95% CI, 74–108) at baseline and 87 mg/dL (95% CI, 61–221) at month 24 (Figure 5C). In patients with low (<74 mg/dL), normal (74–109 mg/dL), or high (>109 mg/dL) blood glucose levels at baseline, no significant change of median blood glucose was observed throughout the study (Figure 5C). In patients with initially low blood glucose levels, a slight increase within the normal reference range was observed.

No significant change of HbA_1c_ was observed in the whole study population from baseline to month 24 (Figure 5B). In patients with an initial HbA1c level ≤5.7%, median HbA_1c_ values significantly increased by 0.3% from 5.1% (95% CI, 4.6–5.6) to 5.4% (95% CI, 5.1–5.8) at month 24 (*p* ≤ 0.012, *f* = 0.92), whereas no change was observed in patients who initially had HbA_1c_ values >5.7% (Figure 5D).

### 3.6. Electrolytes

**Figure 6:**
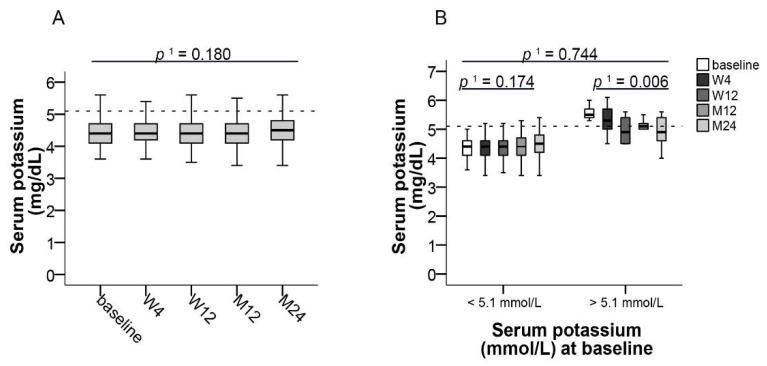
Potassium levels after switching LCPT. Boxplots show median values with 95% CI for (**A**) potassium levels and (**B**) for potassium according to normal or increased values at baseline, both at the baseline visit as well as at weeks 4 and 12 and at months 12 and 24. ^1^ with Greenhouse–Geisser correction; LCPT: once-daily MeltDose^®^ prolonged-release tacrolimus, M: month, W: week.

Median potassium levels were constant during the 24-month observational period in the whole study population (Table 2 and Figure 6A). No change was observed in patients with normal potassium levels (3.5–5.1 mmol/L) at baseline, whereas patients with initially increased potassium levels showed a significant decrease of median potassium by 0.6 from 5.5 (95% CI, 5.3–6.0) to 4.9 mmol/L (95% CI, 4.0–5.6) at month 24 (*p* ≤ 0.006, *f* = 0.74) (Figure 6B).

### 3.7. Trough Levels and Metabolism of Tacrolimus

**Figure 7:**
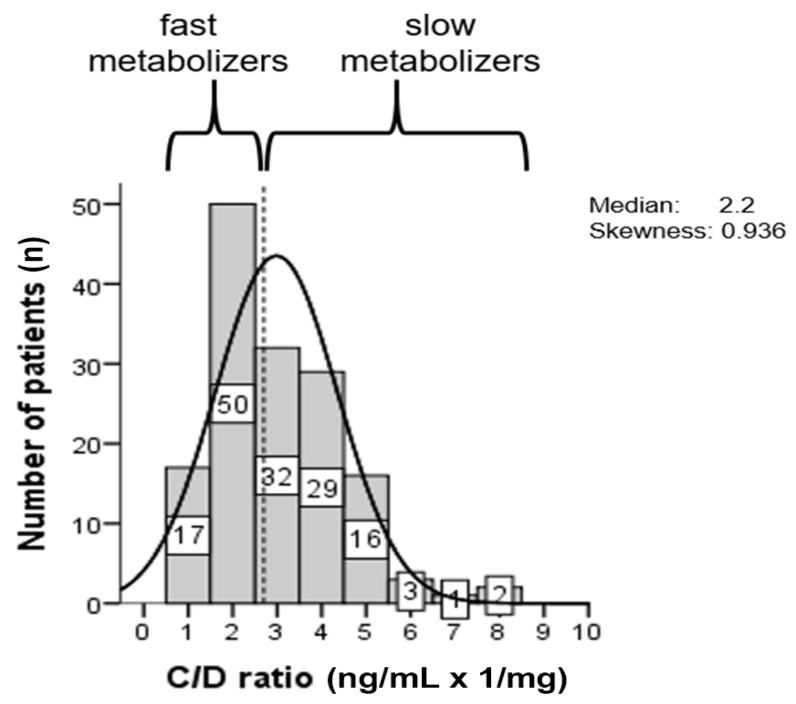
Histogram of the distribution of the tacrolimus C/D ratio (ng/mL × 1/mg). The patients were categorized according to the median value (dashed line) as slow or fast metabolizers [13]. Patients showing a C/D ratio <2.2 were defined as fast metabolizers and patients showing a C/D ratio ≥2.2 as slow metabolizers.

Patients were either considered “fast” metabolizers, if the C/D ratio was below the median of 2.2 ng/mL × 1/mg, or “slow” metabolizers, if the C/D ratio was 2.2 ng/mL × 1/mg or above. According to individual C/D ratios, 67 (44.7%) patients were classified as fast metabolizers and 83 (55.3%) patients as slow metabolizers (Figure 7). 

**Figure 8:**
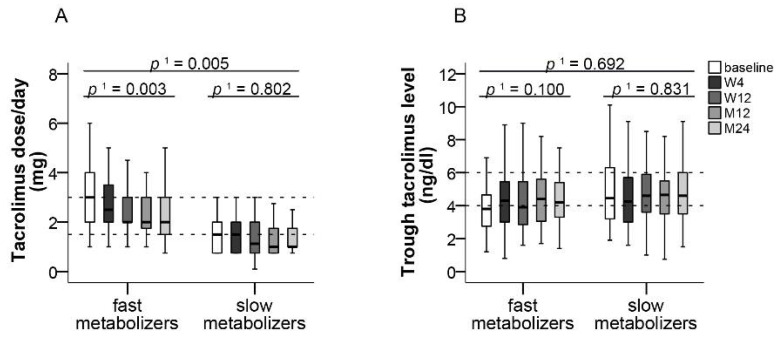
Tacrolimus dose and trough level in slow and fast metabolizers. Boxplots show median values with 95% CI for (**A**) tacrolimus dose and (**B**) tacrolimus trough level according to the categories of slow and fast metabolizers, both at the baseline visit as well as at weeks 4 and 12 and at months 12 and 24. ^1^ with Greenhouse–Geisser correction; M: month, W: week.

In fast metabolizers, median daily tacrolimus dose was significantly reduced by 1 mg (50%) from 3 mg (95% CI, 1–6) at baseline to 2 mg (95% CI, 0.75–6) at month 24 (*p* ≤ 0.003, *f* = 0.62). In slow metabolizers, median daily dose was non-significantly reduced by 0.5 mg from 1.5 mg (95% CI, 0.75–3) at baseline to 1 mg (95% CI, 0.75–4.6) at month 24 (Figure 8A). Median tacrolimus trough levels remained within the target range of 4 to 6 ng/mL throughout the study in both fast metabolizers (baseline: 3.7 mg/dL, 95% CI, 1.2–7.6; month 24: 4.2 ng/dL, 95% CI, 1.4–7.5) and slow metabolizers (baseline: 4.5 ng/dL, 95% CI, 1.6–12.0; month 24: 4.6 ng/dL, 95% CI, 1.5–11.9) (Figure 8B).

### 3.8. Adverse Events

No new safety signals for LCPT were observed during the study, and no severe adverse events were reported throughout the study. In particular, no infection and no hematological alterations (Table 2) were reported. Eleven (7.3%) patients discontinued the study prematurely due to adverse events with no difference between fast and slow metabolizers (*p* ≤ 0.534). Adverse events resulting in treatment discontinuation included asthenia (18.2%) and diarrhea (18.2%), headache (18.2%), facial edema (9.1%) and insomnia (9.1%), hair loss (9.1%), and musculoskeletal complaints (9.1%). For the 11 patients that discontinued the study, we found no association with the etiology of liver disease, gender, or time after LT.

Six (4.0%) patients died during the study due to multiorgan failure, relapse of advanced hepatocellular carcinoma, staphylococcal endocarditis, or suicide. In these patients, death was not considered to be related to therapy.

## 4. Discussion

In this observational study, the switch of immunosuppressive regimens that included IR-Tac to LCPT after liver transplantation was associated with favorable tolerability and graft function within a 2-year observation period.

No new safety signals were observed in our study. No biopsy proven acute rejection (BPAR) was observed during the study period. However, nine patients showed an increase in GGT, which was due to PSC recurrence (*n* = 3/9), co-medication (*n* = 3/9), and ischemic like biliary lesion (ITBL) (*n* = 3/9).

Only 11 patients discontinued LCPT treatment due to side effects, and death in six patients was considered not related to treatment. Thus, we confirm previous findings of good overall tolerability of LCPT in patients after solid organ transplantation [17,23], which is likely due to its smooth pharmacokinetic profile and fewer peak-to-trough fluctuations [19,24]. Accordingly, switching immunosuppressive therapy from IR-Tac or ER-Tac to LCPT has been shown to be associated with significantly lower peak-related toxicity such as tremors, concentration difficulties, headache, and insomnia [23,25].

Switching from IR-Tac to LCPT was associated with a slight increase of median serum creatinine and a higher proportion of patients experiencing GFR reduction at the end of the study. The serum creatinine values stayed within the reference range. The small effect size indicates that the difference between baseline is negligible over time, despite its statistical significance. In previous studies investigating conversion from IR-Tac to LCPT, no renal function data were presented in a 53-week study in liver transplant recipients [18], and in kidney transplant patients a slight but significant increase of mean serum creatinine from 1.56 to 1.61 mg/dL (*p* < 0.05) was observed in a retrospective cohort study in kidney transplant recipients with no changes of GFR [25]. Furthermore, a randomized trial in kidney transplant recipients showed similar estimated GFR (eGFR) between LCPT and IR-Tac patients [26,27], and a systematic review of 10 observational studies did not show differences between IR-Tac and extended-release (ER-)Tac in renal function in adult kidney de novo transplantations [28]. Therefore, our data indicate a minor effect of switching from IR-Tac to LCPT on renal function in liver transplant recipients. Differences may be due to different patient populations included. Recently Einsiedel et al. demonstrated in 61 patients an improvement of the mean GFR (+4.7 mL/min/1.73 m^2^) 12 months after conversion to LCTP in comparison to the control group with standard release TAC [29].

Dysregulated glucose and lipid metabolism are well-recognized complications of organ transplantation and immunosuppression [30]. In particular, glucocorticoids and CNIs have been shown to affect glucose metabolism after solid organ transplantation [31]. Treatment with tacrolimus has been associated with hyperlipidemia, but its occurrence is less severe and less common than with cyclosporine [32,33]. Whereas in our overall study population median total cholesterol levels remained normal, patients who initially were hypercholesterolemic experienced a significant improvement of median total cholesterol after switching to LCPT. Blood glucose levels did not change significantly, irrespective of initial glycemic status, and a slight increase of HbA_1c_ within the normal reference range in patients with normal HbA_1c_ levels prior to switching could be seen. Therefore, throughout our study, only minor changes of glucose and lipid metabolism were observed. However, hypercholesterolemic patients may benefit from switching to LCPT.

Minimization of CNI doses has been identified as one option to reduce CNI toxicity [34,35]. Switching to LCPT has been shown to allow for lower tacrolimus dosing in transplant patients due to better bioavailability. In head-to-head comparisons of renal transplant recipients, LCPT treatment was associated with dose reductions by approximately 30% vs. IR-Tac and by 40% vs. once-daily ER-Tac [17,19]. This is particularly important for patients classified as fast metabolizers who generally require higher IR-Tac doses and who are jeopardized by increased nephrotoxicity [13,36]. In our study, median tacrolimus dose could be reduced by 50% in fast metabolizers, who comprised 50% of the patients, and by 30% in slow metabolizers. Despite dose reductions, no graft rejection occurred within the observation period. Lower tacrolimus dosing may also have contributed to the good overall long-term tolerability of LCPT. In accordance with previous observations, median trough levels of tacrolimus remained within the target range in both fast and slow metabolizers despite dose reductions [25,37]. Identification of fast metabolizers and optimization of tacrolimus formulation could therefore provide a strategy to improve graft survival [38].

The limitations of this study mainly are due to its observational nature and the lack of randomization, which are associated with limited control over data collection. Moreover, our study was conducted at a single center, which limits the generalization of our results to other institutions or populations. On the other hand, the strengths of our study are that adherence was assessed in all patients and that all measurements were performed by the same staff and with the same assays. In addition, by including consecutive patients for conversion to LCPT, selection bias could be avoided.

## 5. Conclusions

In conclusion, our observational study confirms previous evidence of good tolerability and a favorable outcome for patients after switching from IR-Tac to LCPT after liver transplantation. This was associated with dose reductions particularly in fast metabolizers. The dose reduction moreover may contribute to the low occurrence of complications. Clinical parameters of graft function and metabolic profiles essentially remained unchanged, and LCPT did not adversely affect kidney function.

## Figures and Tables

**Table 1:** Baseline patient characteristics.

Variable	
Sex, *n* (%)	
Male	93 (62.0)
Female	57 (38.0)
Age, years, median (range)	55 (18–77)
BMI, kg/m^2^ (range)	24 (4–381)
Switch to LCPT, months (range)	25.5 (17.0–42.5)
Switch to LCPT, *n* (%)	
≤1 year post-LT	33 (22.0)
1–5 years post-LT	76 (50.7)
>5 years post-LT	41 (27.3)
Indications for LT, *n* (%)	
Alcoholic liver cirrhosis	33 (22.0)
Hepatocellular carcinoma	32 (21.3)
Autoimmune liver disease (PBC/PSC/AIH)	24 (16.0)
Non-alcoholic steatohepatitis	10 (6.7)
HBV	8 (5.3)
HCV	7 (4.7)
Cryptogenic liver cirrhosis	6 (4.0)
Acute liver failure	5 (3.3)
Other	25 (16.7)

AIH: autoimmune hepatitis, BMI: body mass index, CRP: C-reactive protein, GGT: gamma-glutamyltransferase, HBV: hepatitis B virus, HCV: hepatitis C virus, LCPT: once-daily MeltDose^®^ prolonged-release tacrolimus; LT: liver transplantation, PBC: primary biliary cirrhosis, PSC: primary sclerosing cholangitis.

**Table 2:** Laboratory parameters at baseline and at 24 months after switching to LCPT.

Laboratory Parameters in Blood, Median (Range)	Baseline	Month 24	*p* Value
Leucocytes, 1/nL	5.6 (2.8–13.7)	5.7 (1.9–11.6)	0.440
Erythrocytes, 1/pL	4.7 (3.1–6.1)	4.7 (3.1–6.5)	0.747
Hematocrit, L/L	0.39 (0.22–0.47)	0.39 (0.26–0.94)	0.099
Hemoglobin, g/dL	13.1 (4.4–17.2)	13.2 (8.2–17.2)	0.211
Thrombocytes, 1/fL	177 (58–488)	181.0 (51–417)	0.167
Sodium, mmol/L	140 (133–149)	140 (130–147)	0.195
Potassium, mmol/L	4.4 (3.6–6.0)	4.5 (3.4–6.8)	0.106
Magnesium, mmol/L	0.74 (0.58–0.96)	0.74 (0.42–0.92)	0.022
Proteinuria, g/L	9.1 (4.9–60.7)	8.3 (4.9–287)	0.150
Serum creatinine, mg/dL	1.1 (0.63–4.34)	1.1 (0.64–4.52)	0.006
Urea, mg/dL	18.0 (5.0–68.0)	18.0 (6.0–84.0)	0.157
Uric acid, mg/dL	6.1 (2.6–12.1)	5.9 (2.6–56.0)	0.229
AST, U/L	23 (9–98)	21 (8–81)	0.109
ALT, U/L	23 (8–141)	24 (6–152)	0.561
AP, U/L	105 (44–937)	91 (39–317)	0.002
GGT, U/L	26 (6–891)	25 (7–917)	0.448
Albumin, g/dL	4.3 (3.4–5.0)	4.4 (3.6–5.0)	0.210
CRP, mg/dL	0.3 (0.3–3.5)	0.3 (0.3–4.2)	0.482
Total cholesterol, mg/dL	193 (83–318)	189 (89.0–360.0)	0.202
Triglycerides, mg/dL	133 (37–677)	129 (39–709)	0.560
Glucose, mg/dL	94 (54–258)	93 (52–244)	0.257
HbA_1c_, %	5.6 (4.4–10.5)	5.7 (4.3–9.0)	0.659
Total bilirubin, mg/dL	0.5 (0.2–2.8	0.6 (0.2–2.8)	0.887
Direct bilirubin, mg/dL	0.2 (0.1–2.3)	0.2 (0.1–1.9)	0.150

ALT: alanine amino transferase, AP: alkaline phosphatase, AST: aspartate amino transferase, BMI: body mass index, CRP: C-reactive protein, GGT: gamma-glutamyltransferase.
